# Exosomes as a Surrogate Marker for Autophagy in Peripheral Blood, Correlative Data from Phase I Study of Chloroquine in Combination with Carboplatin/Gemcitabine in Advanced Solid Tumors

**DOI:** 10.31557/APJCP.2019.20.12.3789

**Published:** 2019

**Authors:** Nagla Abdel-Karim, Ola Gaber, Ihab Eldessouki, El Mustapha Bahassi, John Morris

**Affiliations:** *Department of Hematology –Oncology, University of Cincinnati, U S A. *

**Keywords:** Autophagy, cancer, exosomes, biomarker, western blotting, treatment

## Abstract

**Background:**

Autophagy is a catabolic process, utilized constitutionally by body cells to recycle nutrients and to remove unwanted/damaged intracellular constituents. It is enhanced during periods of stress, such as starvation and hypoxia, aiding in cell survival and it is linked to major cellular processes, such as apoptosis and antigen expression. The process has been extensively studied in vitro models or tumor tissue samples with rare application on human subjects.

**Methods:**

Plasma samples from 24 advanced solid tumor patients were collected at different time points before and after chemotherapy. Their exosomes were isolate and blotted for microtubule-associated protein-1 light chain-3 (LC-3B) protein as a marker for autophagy. All the subjects received a standard chemotherapy regimen of carboplatin- gemcitabine with chloroquine (CQ)/ hydroxychloroquine (HCQ) in chronic doses throughout their treatment period as an autophagy modulator. CQ/HCQ was given in 50 mg increments as guided by their tolerability to treatment.

**Results:**

A total of 267 plasma samples were obtained for the 24 patients and processed. Each sample corresponds to a single time point. The first group included 6 patients, all received 50 mg of CQ with chemotherapy. LC-3B I was detected in their isolated exosomes, while LC3-BII was not detected in their samples. The second cohort of patients included 3 subjects who re-ceived 100mg of HCQ. They demonstrated both LC3-BI and II on day 15 after chemotherapy in one patient, and on third cycle after 24 hours in the second patient. The third cohort included 3 subjects who received 150 mg of HCQ. All cases demonstrated LC3-BI and II on first cycle of treatment after less than 24 hours. The last cohort included 8 subjects, who received a fixed dose of 100 mg of HCQ with treatment. In this cohort, we were able to detect both LC3-B isoforms on advanced time points of second and third cycles.

**Conclusion:**

Detection of autophagy protein LC3-B in exosomes serves as a dynamic method to monitor autophagy. It can be utilized to study the effects of anti-neoplastic agents on autophagy and mechanisms of drug resistance, however, to standardize our results a larger specimen of patients should be included.

## Introduction

Autophagy is a self-degradative process that occurs in eukaryotic cells to maintain protein he-mostasis and recycling of organelles. It is a dynamic process that occur constitutively and in re-sponse to cellular stressors such as cell starvation and hypoxia (Kroemer et al., 2010). Three forms of autophagy occur in mammalian cells, a) micro-autophagy, describes the simple process of lysosomes directly engulfing small cytosolic components (Mijaljica et al., 2011), b) chaperone-mediated autophagy (CMA) which is a more selective process in which cytosolic proteins that will be degraded are marked with KFERQ-like pentapeptide motifs that are specifically recognized by chaperone hsc70, and on the lysosomal surface LAMP-2A acts as a receptor to hsc70 and engulfs these proteins inside the lumen to be degraded(Arias and Cuervo, 2011), and finally c) macro-autophagy: referred to as autophagy, and the process referred to in our study.

Autophagy has been widely studied in vitro to understand its dynamics and interactions with other cellular processes including cancer. It has been pointed at as both a culprit that enables cancers cells to survive (Hashimoto et al., 2014) and evade antitumor agents (Chen et al., 2014a), and a physiological mechanism that helps with protection of normal cell integrity and initiation of apoptosis in occasions of abnormal transformations (Mizushima et al., 2008). Some reports considered autophagy as one way through which anticancer agents can achieve their effect (Notte et al., 2011; Sui et al., 2013; Ranjan and Srivastava, 2016), others went to the con-clusion that it may actually have a direct effect on tumor behavior (Guo et al., 2013; Peng et al., 2013).

Even though, its role in cancer development, progression, and metastases has been widely described with a vast amount of data, most of these data were obtained from in vitro and mice models (Kuma et al., 2004; Komatsu et al., 2006; Mizushima et al., 2008; Zou et al., 2013; Chen et al., 2014a; Lowry et al., 2015). The main focus is to establish connection to other process by monitoring effects on their biomarkers. Seldom these data were translated into human subjects to verify its applications. 

A rather brief description of the process entails its initiation through formation of an isolation membrane, termed phagophore. Phagophore origin is not well defined. Some studies suggested an origin from specialized regions in the endoplasmic reticulum, others suggest that Golgi apparatus and mitochondria. Phagophores start engulfing organelles and macromolecules from the cytoplasm, thus evolving into another structure known as the autophagosome. This is a double membrane vacuole with a size range from 300 to 900nm. It requires maturation through fusion with early and late endosomes (Dunn, 1994; Berg et al., 1998; Glick et al., 2010; Stolz et al., 2014), forming a hybride organelle called the amphisome. This the final structure which can fuse with lysosomes for degradation of their contents (Deretic, 2008). However, it was reported that autophagosomes can fuse directly with lysosomes (Liou et al., 1997). While this sequence of ongoing events is taking place, exosomes are formed and released by the cell, and their release is a dependent process that is directly linked to autophagy. It was suggested that inhibition of autophagy, increase exosomal release and vice versa (Fader et al., 2007).

Exosomes are a form of extracellular vesicles with a size ranging between 30-100nm in diame-ter(Simons and Raposo, 2009). It is believed that they originate from intraluminal vesicle (ILV) inside the late endosomes or the multi-vesicular bodies (MVB) by inward budding of their membranes. When the MVB fuse with the cell membrane and release its contents in the extracellular environment, which are the exosomes (Fader and Colombo, 2006; Bellingham et al., 2012). There was a dominating trend that believed that exosomes are the cell way to get rid of unwanted structures (Fader and Colombo, 2006; Zhang and Schekman, 2013; Baixauli et al., 2014). However, the precisely tailored exosome structure (Smith et al., 2015), and the specificities of the mechanisms involved in selective loading of its contents argue against it. Even though it is plausible that this can occur as part of the universal picture regarding the exosomal endocytic origin. It exosomes have been implicated in metastases, abnormal DNA changes and intercellular communication (Kucharzewska and Belting, 2013). This is thanks to its content of specialized species of microRNAs (miRNA)(Kuma et al., 2004; Lowry et al., 2015; Prathipati et al., 2017). 

Previous reports have suggested the use of exosomes as biomarkers for autophagy in breast cancer cell lines (Liou et al., 1997). And the concept is interesting, since exosomes secretion and autophagy are both reflect the dynamicity of the cellular process. Hereby, we tested this hy-pothesis in a population of 24 advanced solid tumor patients (Karim et al., 2017, 2018; Eldessouki et al., 2018; Abdel Karim et al., 2019; Abdel Karim et al., 2019; Gaber et al., 2019). The patients were treated with con-ventional gemcitabine-carboplatin regimen. During their treatment period they received daily dose of chloroquine/hydroxychloroquine, as an autophagy modulator. The effect on the changes on autophagic activity was monitored through exosomes isolated from the patient plasma at different time points. The exosomes LC3-B content was demonstrated through western blotting.

## Materials and Methods

*Study population*

This study included 24 patients who were recruited in 4 cohorts, cohort 1 (n=6), cohort 2 (n=3), cohort 3 (n=3) and extension cohort (n=8). All patients were histologically diagnosed with ad-vanced solid. All subjects provided a written informed consent before treatment in accordance with the Declaration of Helsinki, and the study protocol was approved by the Institutional Re-view Board of the University of Cincinnati Hospital. The subjects enrolled have failed their previous lines of treatment and the proposed chemotherapy regimen (Carb/Gem) was considered a standard of care 

*Cell Culture*

We used four cell lines in this study, human glioblastoma cell line (U251), human pancreatic carcinoma cell line (MiaPaCa2), adenocarcinoma human alveolar basal epithelial cells (A549) and human embryonic kidney cells (HEK293). The variety was intentional to insure matching results regardless of baseline autophagic activity of the cell line. These cells were maintained in DMEM media, supplemented with 10% fetal bovine serum (FBS), antimycotic, antibiotic and L-glutamine. At 40% confluency, the culture dishes were washed twice with PBS and the media replaced with 10% exosome depleted FBS (dFBS) DMEM media (50 ml EXO-FBSTM Exosome-Depleted FBS Media, System Biosciences #EXO-FBS-50A-1) and incubated to settle for 24 hours. After 24 hours, the culture dishes were washed with PBS and new dFBS DMEM media was added and treated with chloroquine diphosphate salt (Sigma-Aldrich, UK) and incubated. Incubation period was 48 hours for western blotting on HEK293 plates and 72 hours for western blotting on U251. The discrepancy in the incubation time was attributed to the confluency of the plates, CQ concentration used and the number of exosomes expected to be secreted by the cell line. The media was collected and used for exosome extraction directly after the incubation time is over.

*Exosome extraction*

*1- From Patients Plasma*

Patients’ blood samples were collected at the mentioned time points and span down at 1500 g for 15 minutes. The upper phase (plasma) was collected in new tubes and stored at -80 degrees till us. Exosomes extraction was done using an exosome extraction reagent (Total Exosomes Precipitation Reagent from plasma, Invitrogen by Thermo-Fisher Scientific Ref: 4484451) following manufacturer’s instructions, then, suspended in PBS and stored at -80^o^C.

*2- From Cell Culture Media*

Exosomes were extracted from freshly collected cell culture media using exosome precipitation reagent (50ml Total Exosome Isolation from cell culture media, Invitrogen^Tm^ by Life Technologies^TM^, ref# 4478359) following manufacturer’s instructions. Extracted exosomes were suspended in PBS and stored at - 80^o^C. The method for patient extraction is described in full details here (Gaber et al., 2019).

*Western blotting*

Detection of LC3b expression in the isolated exosomes was done using western blotting following standard protocols. LI-COR detection was used to scan the membranes. LC3B protein detection was achieved by anti-LC3B rabbit monoclonal antibody (Cell Signaling Inc., catalogue #2775, USA). CD9 was used as a loading control was blotted using rabbit monoclonal antibody (#3700) from Cell Signaling Technology Inc. All western blots were run on 4-15% gradient gels after estimating and unifying samples protein content by BCA.

*Flow cytometry*

HEK293 cells were plated in 10 cm culture dishes in 10% FBS DMEM media. The media was re-placed with dFBS DMEM media once a 40% confluency is reached and incubated for 24 hours. Drugs as single agents, Gem at 20uM concentration (Gem20), CQ at 10uM (CQ10) and 20uM (CQ20) concentrations, and in combinations: Gem+CQ10 and Gem+CQ20 were used to treat the plated HEK293 cells in fresh dFBS DMEM media for 16 hours. Both the cells and the media were collected, spinned down, then washed and finally stained with propedium iodide (PI) and annexin V before running the samples. Cell cycle analysis was performed using fresh cells on a FACS Calibur (Becton Dickinson) after incubation with 25 μg/ml of PI. Cell cycle phases were analyzed with the CellQuest-Pro software program (Becton Dickinson). 

## Results

*In vitro experiments*

We started by measuring the basic autophagy activity in the four cell lines that we used in this study through extraction of the exosomes from the cell culture media and blotting them for LC-3B. We used time points that corresponds to the post treatment time points at which the samples were withdrawn from the patients. These time points were: 1h, 2h, 4h, 6h and 24h. Since these cells were not treated with any agents, we calculated the time points from the point when fresh exosome free media was added to the cell culture dishes at 60% confluency. Blotting the cells showed equal expression for LC3-BI and II for all cell lines at all time points, however, the bands were most clear at 24h (Figure 1.1b). The cell culture media was collected at the same time points from the same plates to blot for the same protein. While LC-3BI was vaguely detected at time points beyond the 24h point that we originally set, and LC-3BII was detected at 48 hours. However, incubation after 24 hours increased the amount of cellular debris and necrosis. We concluded that we can use the 24h time point as a reference at which the optimum number of exosomes are being released without treatment. The U251 cells showed more expression of LC3-B isoforms compared to other cell lines which indicated a higher basal autophagic activity (Figure 1.2b).

Then we used CQ treatment on the mentioned cell lines to monitor the effective changes in LC-3B expression in the cells and exosomes compared to baseline. We started with different concentrations 25uM, 50uM, 100uM and 120uM, we found that at the set time point of 24h, the 100uM concentration produces enough inhibition of cellular confluency without cellular necrosis. At a concentration of 100uM of CQ, the exosomes extracted from cell culture media of HEK293 cells showed LC-3BII bands at 24h, however, no bands were detected for LC-3BI (Figure 1.1a), this is compared to no bands at 24h in the untreated samples. This observation can be explained by increased exosomes release as a response to the autophagy modulatory effects of chloroquine. 

**Figure 1:**
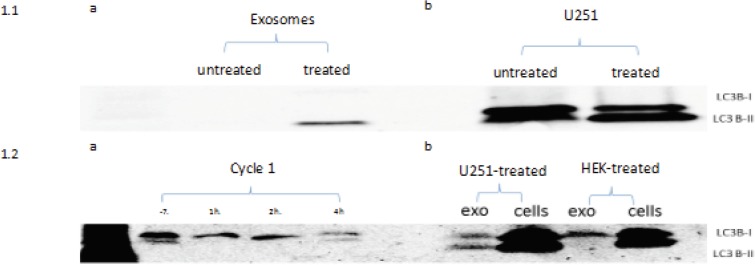
Showing the Results of LC-3B Blotting. 1.1a exosomes extracted from the cell culture media of HEK293 cells, 1.1b U251 cells, 1.2a cycle 1 of patient who received CQ 150 mg starting 1 week before chemotherapy and after collection of day -7 sample collection, and 1.2b exosomes collected from U251 cell culture media and U251 cells, and exosomes from cell culture media of HEK293 cells and HEK293 cells

Figure 1: showing the results of LC-3B blotting: 1.1a exosomes extracted from the cell culture media of HEK293 cells, 1.1b U251 cells, 1.2a cycle 1 of patient who received CQ 150 mg starting 1 week before chemotherapy and after collection of day -7 sample collection, and 1.2b exo-somes collected from U251 cell culture media and U251 cells, and exosomes from cell culture media of HEK293 cells and HEK293 cells. 

**Figure 2:**
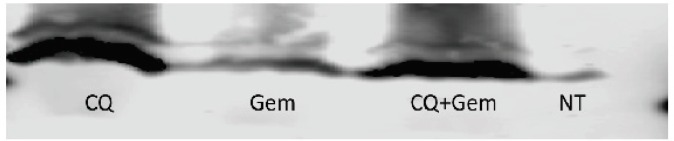
showing Results of LC-3B I and II Blotting in Exosomes Extracted from the Cell Culture Media of MiaPaCa 2 Cell-Line

Since the treatment regimen given to the patients included an autophagy stimulator (Chen et al., 2014a), gemcitabine (Jiang et al., 2017) and CQ which inhibits the final stages of autophagy, we wanted to test their effect on LC-3B expression on released exosomes. We set 4 types of treatments for MiaPaCa2 cells including CQ 20uM, Gem 20uM and a combination of both. These concentrations were decided based on the degree of confluency and necrosis after 24h of treatment. We found out that the combination treatment produced by far a thicker band for LC-3BII isoform compared to the Gem treatment only. Although the band was slightly thin compared to CQ treatment, but the effect of the combination treatment was evident (Figure 2). This experiment showed a clear difference in the outcome based on the thickness and intensity of the bands, between autophagy inhibitor and an autophagy inducer. The same results were similar in HEK293 and U251 cells.

**Figure 3:**
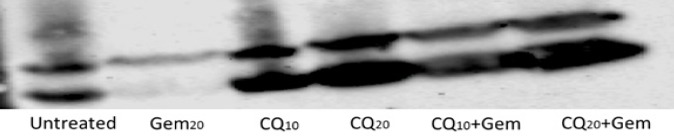
Showing Results of LC-3B Blotting of Exosomes Extracted from the Cell Culture Media of U251 Cells. Gem treatment resulted in marked reduction in the intensity of the bands, while reducing the dose of CQ resulted in thinner bands while the intensity of the bands were the same

To further investigate this point, we increased the treatment time to 36h and the effect of inhibition and induction was further augmented in the form of fainter bands with Gem and thicker bands with inhibition and combination treatment. CQ inhibition seems to overcome Gem inducing effect since at the same dose combination treatment resulted in equal effects, however, reducing the dose of CQ for treatment resulted in thinner bands (Figure 3). 

*In vivo experiments*

A total of 261 samples were obtained for the 24 participants. The time points for the patients were more extended; day -7 (7 days prior to administration of the first cycle), 48h, 72h, day 8 and day 15 samples were also collected. These time points varied from one patient to another since some patients did not tolerate the treatment or they dropped out from the study. The exact details of the patient data will be reported in a separate report.

**Figure 4:**
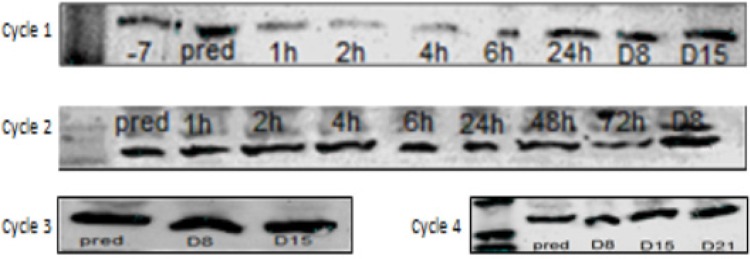
Showing LC-3B Blotting of Exosomes Extracted from the Plasma of a Patient who Received 50 mg of CQ with Treatment. The faint bands post-treatment can be observed only in the first cycle. The second, third and fourth cycles showed LC-3B II bands with increased intensity

The first cohort of patients included 6 cases who received Carb/Gem at standard doses. CQ/HCQ was started at 50 mg fixed daily dose 7 days before treatment and continued throughout their course of treatment. Exosomes did not show expression of LC-3B II in the western blotting for most cases, however, exclusion of effect for CQ/HCQ at this dose level cannot be excluded since most of the enrolled cases did not received a full course of Carb/Gem and they were dropped out of the study. One patient complete the full course of treatment and we were able to detect LC-3B II starting from the second cycle. The band was very clear on day 8 of the second cycle and ultimately became the predominant band on third and fourth cycles while LC-3BI faded away and became almost nonexistent (Figure 4). 

**Figure 5:**
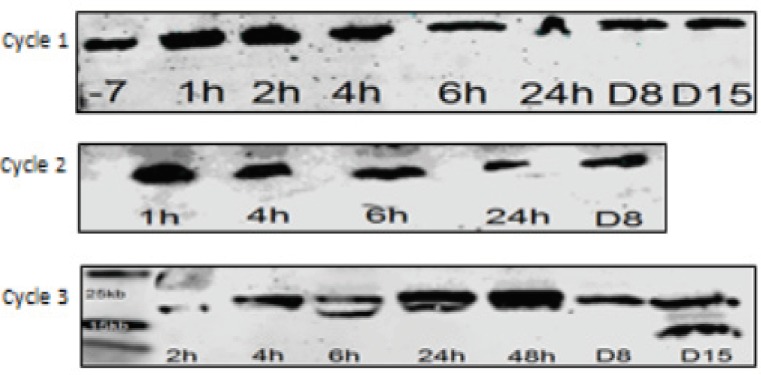
Showing LC-3B Blotting of Exosomes Extracted from the Plasma of a Patient who Received 100 mg of HCQ. The patient showed no expression of LC-3B II at baseline. LC-3b II protein was expressed on day 15 of the third cycle

The second cohort included 3 cases who received 100 mg of CQ/HCQ throughout the treatment period and more patients remained longer in the study. One patient showed bands on an earlier time point during his treatment course with LC-3B II evident on day-7 and after 4 hours of the first cycle of the first patient (Figure 1.2a), while another patient expressed LC-3B II only on his third cycle on day 15 (Figure 5). This discrepancy in our findings can be explained by different baseline activity in both patients. the first patients had *LC-3B II* expression at baseline, which was lacking in the second patients and hence the variation in the onset of response. 

**Figure 6:**
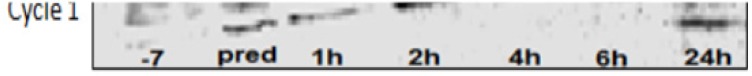
Showing LC-3B Blotting of Exosomes Extracted from the Plasma of a Patient who Received 150 mg of HCQ. LC-3B II band can be seen at 24h time point. This the earliest time point post-treatment that LC-3B II was detected

The third cohort included another 3 patients who received 150 mg of CQ/HCQ during the study period. Although some patients lacked *LC-3B II* expression at baseline, they expressed LC-3B bands on the first cycle. In Figure 6* LC-3B II* was expressed at 24h, another case expressed the protein at 24h and the last patient expressed it on day 15. 

Since, 2 patients were not able to tolerate the 150mg regimen in cohort 3, 8 more cases were enrolled under the 100mg regimen schedule to validate the results. Like the previous cases, the patients expressed LC-3B II starting from the late time points of the second or the third cycle. This validated our conclusion that the effects of CQ/HCQ are not only dose dependent but also have a cumulative effect.

*Necrosis or apoptosis*

**Figure 7:**
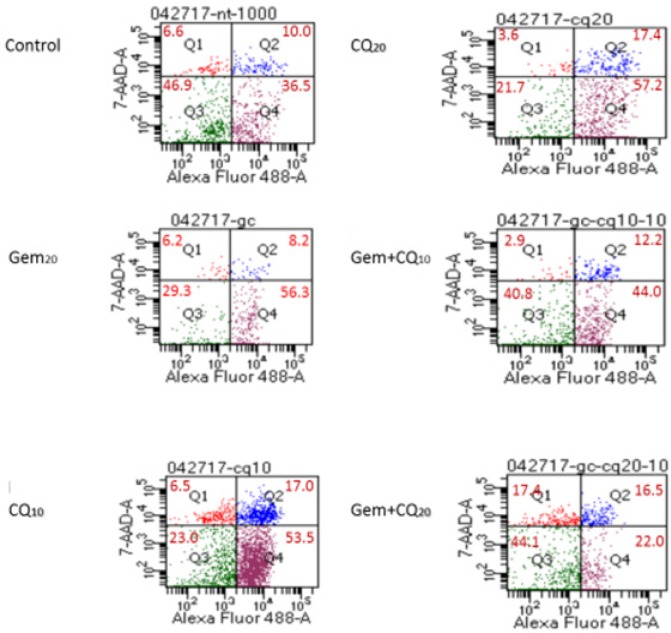
Showing the Annexin V Assay for the MiaPaCa 2 Cells Treated with Gem and CQ at Different Concentrations. Compared to controls and Gem treatment, CQ resulted increase in apoptotic and pre-apoptotic cell percentage. Increased CQ concentration resulted in marked increase in necrosis

Since the in vitro experiments have shown more cellular death with combination treatment at 24h of treatment we concluded that the regimen kills tumor cells at a higher rate than each drug alone. To investigate this phenomenon, we ran an annexin V assay to verify if the effect is due to necrosis or apoptosis. HEK295 cells were treated with CQ 10uM, CQ 20uM, Gem 20uM and Gem with either CQ 10uM or 20uM. The cells were stained with annexin V. The results showed that compared to control all treatments had less live cells. Treating the cells with CQ 20uM showed as low as 21.7% live cells compared to 49.7% for control. The combination treatment showed higher percentage of live cells at 40.8% and 44.1 % for Gem+ CQ10 and Gem+CQ20 respectively. Combination therapy showed high percentage of apoptotic and pre-apoptotic cells. The increase in CQ concentration with Gem also played a role in increasing the number of necrotic cell population to 17.4% compared to 6.6% for controls and 2.9% for the Gem+CQ10 (Figure 7).

## Discussion

In this study we investigated a new method to monitor autophagy in human plasma. Plasma samples were obtained from 24 advanced solid tumor patients at different time points and planned according to their chemotherapy regimen schedules. The patients received either CQ or HCQ during the full course of their treatment at fixed daily dose and each cohort received increasing increments of 50 mg. The starting dose was 50 mg and the time points were obtained 1 hour before treatment administration and after administration of their scheduled dose at 1h, 2h, 4h, 6h, 24h, 48, 72h and on days 8 and 15. Exosomes were isolated from the patients’ plasma and blotted for autophagy protein LC-3B using increased transformation of LC-BI isoform to LC-3B II expression as an indication of increased autophagic activity(Tanida et al., 2005; Koukourakis et al., 2015). We found that the patients had a different patterns of baseline LC-3B expression before treatment. Patients who expressed LC-3B II before treatment, did not express it in the early time points after receiving chemotherapy. The autophagy modulating effect of CQ/HCQ arguably inhibits the late stage of the process when the late endosome fuses with lysosomes (Ganguli et al., 2014; Maes et al., 2016), while Gem effect is increasing the turnover of the process (Chen et al., 2014b). So, we hypothized from our results that together both agents increased the number of released exosomes facilitating their detection in peripheral blood. However, this theory needs further investigation and exclusion of many other variables including other agent used, which is carboplatin, and the effect of other medications administered for other morbidities. Our in vitro studies had shown a clearer distinction between autophagy induction by Gem and inhibition by CQ. The inhibitory effect was manifested in the form of increased LC-3B II expression and changing the dose affected the band thickness in western blotting rather than its intensity. Meanwhile induction by Gem produced thick but faint bands on western blotting. We assumed that the stimulation of the process resulted in increased conversion of LC-BI to LC-BII isoform, however, the CQ/HCQ resulted in accumulation of the protein rather than its degradation due to inhibition of lysosomal fusion. Annexin V assays showed increase in apoptotic and pre-apoptotic cells compared to control with single and double agent treatments using Gem and/or CQ/HCQ. The percentage of necrosis increased with higher CQ dosage, which might suggest that the increased dosage have a higher toxic effect on cells that is yet to be defined. 

Many studies have suggested the implication of autophagy as means by which tumors develop resistance to therapeutic effects of anti-neoplastic agents (Peng et al., 2013; Zou et al., 2013; Liang et al., 2014). Also many studies suggested that autophagy is affect by many anti-neoplastic agents (Chen et al., 2014b). The extent of implication of this pivotal process has been linked to other vital process of the body beyond cancer therapy (Cuervo et al., 2005; Kaur and Debnath, 2015). And with emergence of immunotherapy such process needs to be further understood since autophagy plays a major role in cellular antigen expression (Witko-Sarsat and Codogno, 2013; Shibutani et al., 2015). And it has been implicated in evading immune surveillance (Janji et al., 2016). Thus, can be related to various predictive and prognostic markers(Darwish et al., 2016; Abdel Karim et al., 2018; Magdy et al., 2018, 2019; Rahouma et al., 2019). However, these studies have always been in vitro experiments or done using murine models. Nowadays with the advanced techniques in obtaining and analyzing blood based biopsies (Abdel Karim et al., 2018; Gaber et al., 2018), developing biological markers to monitor such pivotal process in patients becomes necessary to evaluate if our understanding to these concepts applies to the heterogenous nature of our patients and the dynamic changes of the disease. 

LC-3B conversion from LC-3B I to II has been used as an indicator for autophagy since it measures the dynamicity of the process by reflecting the turnover of autophagosome fusion with lysosomes (Tanida et al., 2008). However, increased expression of both isoforms is used to measure the activity of both autophagy inducers and inhibitors (Sharifi et al., 2015; Redmann et al., 2017). Although this method is biologically explained (Tanida et al., 2008; Koukourakis et al., 2015), but there was no explanation on how to differentiate between both actions and unless other quantification methods are used, both actions can be interpreted similarly on western blotting. Our in vitro results had showed different picture for each when LC3-B was measured in exosomes instead of cells. This conclusion can serve as simple and cost-effective method to track stimulus effect on autophagy by interpretation of t western blotting compared to well known controls.

## Ethics approval and consent

All subjects that participated in the study were consented before the study.

All necessary approvals were granted by the IRB of the University of Cincinnati.

## Abbreviations

LC3-B: microtubule-associated protein-1 light chain-3 

BCA: Bicinchoninic acid assay

Carb: Carboplatin

CQ: Chloroquine

CQ10: Chloroquine 10uM

CQ20: Chloroquine 20uM

dFBS: Exosome depleted FBS

ESCRT: Endosomal sorting complexes required for the transport 

FBS: Fetal bovine serum.

Gem: Gemcitabine 

HCQ: Hydroxychloroquine

ILV: Intraluminal vesicle 

kDa: Kilo Dalton

LC3B: Microtubule-associated protein-1 light chain-3

MVB: Multi-vesicular bodies

PI: Propedium iodide

WB: Western blotting

## Conflict of interest

The authors whose names are listed certify that they have NO affiliations with or involvement in any organization or entity with any financial interest (such as honoraria; educational grants; participation in speakers’ bureaus; membership, employment, consultancies, stock ownership, or other equity interest; and expert testimony or patent-licensing arrangements), or non-financial interest (such as personal or professional relationships, affiliations, knowledge or beliefs) in the subject matter or materials discussed in this manuscript. 

## Funding

The research was funded by an internal grant from the University of Cincinnati.

## Authors Contribution

Ola Gaber: Research, data collection, writing.

Ihab Eldessouki: research, writing.

El-Mustapha Bahassi: supervision of correlative studies.

John C. Morris: Clinical management, supervision.

Nagla Abdel Karim: Clinical trial rational, supervision.
